# Bent Fiber Sensor for Preservative Detection in Milk

**DOI:** 10.3390/s16122094

**Published:** 2016-12-09

**Authors:** Omer Galip Saracoglu, Sekip Esat Hayber

**Affiliations:** 1 Department of Electrical and Electronic Engineering, Erciyes University, Kayseri 38039, Turkey; 2 Department of Electronic and Automation, Kaman Vocational College, Ahi Evran University, Kirsehir 40300, Turkey; sehayber@ahievran.edu.tr

**Keywords:** bent fiber sensor, plastic optical fiber, concentration sensing, refractive index, milk

## Abstract

A fiber optic sensor sensitive to refractive index changes of the outer region of the fiber cladding is presented. The sensor uses bent plastic optical fibers in different bending lengths to increase sensitivity. Measurements were made for low-fat milk, the refractive index of which is altered by some preservatives such as formaldehyde, hydrogen peroxide, and sodium carbonate. Concentrations of the preservatives in the milk were changed between 0% and 14.3% while the refractive indices occurred between 1.34550 and 1.35093 for the minimum (0%) and maximum (14.286%) concentrations of sodium carbonate, respectively. Due to bending-induced sensitivity, the sensor is able to detect refractive index changes less of than 0.4%. The results show that there is excellent linearity between the concentration and normalized response of the sensor.

## 1. Introduction

Technological developments have made every area of our lives easier. One of the developments has taken place in the food industry because biosensor-based sensing technologies offer advantages in industrial processes and also in health and chemical ones [1]. In order to ensure safety and hence to increase food quality, the monitoring of contaminants and impurities such as preservatives in natural and daily foods has become a major interest [2,3,4]. Milk and products made from milk have a special place not only in our daily nutrition but also in our health. Because of the fact that they can carry harmful bacteria and microorganisms, they can spoil the products and make people sick. For this reason, many researchers have attempted to develop biosensors for detecting compositions and some contaminants such as enterotoxins, antibiotics, bacteria, aflatoxins [5,6,7,8,9] and monitoring some critical parameters such as the cutting time, pH, temperature, and enzyme concentration [10,11,12,13,14,15,16] in milk and its products.

In order to produce quality and healthy products and to reduce infection risks, the purity of milk must be monitored at every stage of the process [17,18,19]. This is because researchers have used optical biosensors, especially by using light-matter interactions at both the molecular and bulk levels, for detection or monitoring of the critical parameters in milk and its products [8,10,20]. Optical biosensors utilize light-matter interactions at different levels and they have interesting features and abilities when compared to conventional electronic sensors such as immunity to electromagnetic interference, high sensitivity and selectivity, label-free detection, and ability for remote sensing [21,22]. 

There are some successful examples of fiber optic biosensors in open literature. Hao et al. used a fluorescence-based fiber optic probe to detect melamine in dairy samples such as liquid milk, yoghurt and baby formula milk [20]. They achieved detection with high sensitivities ranging from 12.62 to 284.18 µg/L. Castillo et al. determined the milk cutting time with a fiber optic sensor by measuring backscattered light. They also monitored milk coagulation with the sensor [10]. The evanescent field created by the light passing through the fiber with total internal reflections can also be used for sensing purposes [20]. A plastic optical fiber (POF) biosensor was proposed by Wandermur et al. to detect *Escherichia coli* [8]. In such sensor designs, a bent or tapered sensing area offers better sensitivities [8,23,24,25]. In addition to fluorescent or absorption-based techniques, Jain and Sarma used the light-scattering technique to modulate UV-vis radiation for the online analysis of milk [4] and Liu et al., used near-infrared spectroscopy for similar purposes [5].

In this work, we designed and implemented a fiber optic sensor made of plastic optical fibers to detect some preservatives such as formaldehyde, hydrogen peroxide, and sodium carbonate. In order to ensure better interactions, we coiled the fibers with different turns. As a result, our simple design has been successfully able to detect the preservatives at concentrations of less than 5%.

## 2. Materials and Methods

### 2.1. Theory

**Figure 1:**
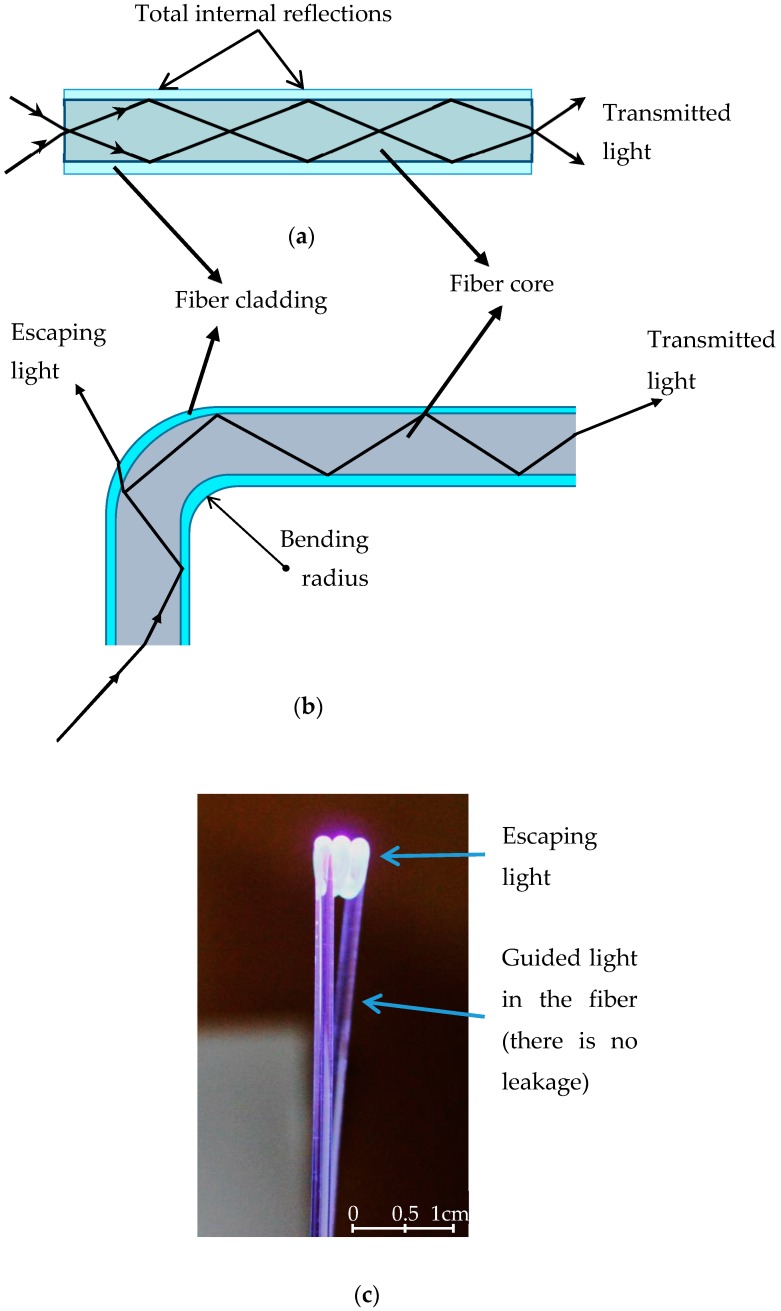
Light guiding in a multimode fiber. (**a**) Total internal reflection; (**b**,**c**) escaping light because of the bending.

Power loss in optical fibers depending on bending is one of the signal attenuation mechanisms. In a multimode plastic optical fiber (POF), the light is guided by total internal reflections that occur in core-cladding interface of the fiber as shown in Figure 1a [26]. The guided light is tightly confined by the cladding at the interface while it passes through the fiber. However, a considerable part of the light leaks out the cladding as evanescent wave when the fiber is bent with a critical radius (Figure 1b). If the fiber is bent the smaller radius than the critical radius, the guiding condition in the fiber is degraded and a considerable part of the light escapes out the fiber (Figure 1c). While this signal loss can be considered as a drawback in optical fiber communication, it can be utilized as a useful tool for sensing purposes [24,25,26,27,28,29,30]. In this work, although there were successful samples of U-Shaped bent fiber sensors [31,32], the sensing area was formed by our designed coil shaped probe for the higher sensitivities.

The schematic in the Figure 1 is the main principle of the bent fiber sensors. The sensitivity can be adjusted depending on the bending radius and then, an amplitude modulation can be achieved by changing refractive index of the medium surrounding the bent area. Moreover, this principle can be used for concentration sensing because the refractive index of the surrounding medium is related to the concentration. 

**Figure 2:**
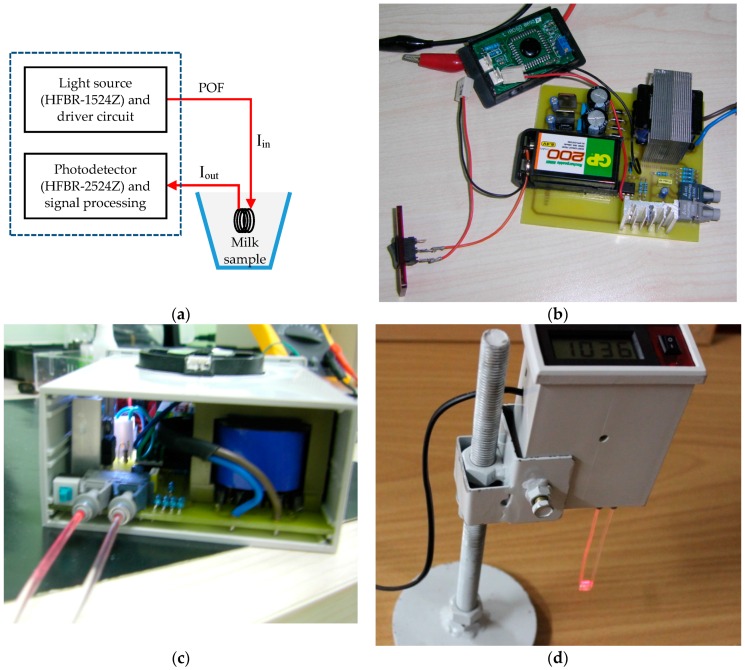
Sensor setup. (**a**) Schematic view of the setup; (**b**) printed circuit board placement; (**c**) inside view; (**d**) the final design ready for immersion into the milk sample.

Our main goal in this work is to design a sensor system being capable of detecting the possible impurities in milk for every stages of the milk processing. So, we designed and implemented an optical sensor system to detect some contaminants such as formaldehyde, hydrogen peroxide, and sodium carbonate in low-fat commercial milk. The sensor uses POFs for both the light guiding medium and the sensor probe. Light source and photo detector of the sensor is a 660 nm LED (typical output power is 0.158 mW at 25 °C) and a photodiode-IC receiver (sensitivity is 0.135 mW at 25 °C), respectively (the transmitter and the receiver are from Avago Technologies, HFBR-1524Z and HFBR-2524Z, respectively [33]). The details of the sensor setup are shown in Figure 2.

### 2.2. Sensing Probe

**Figure 3:**
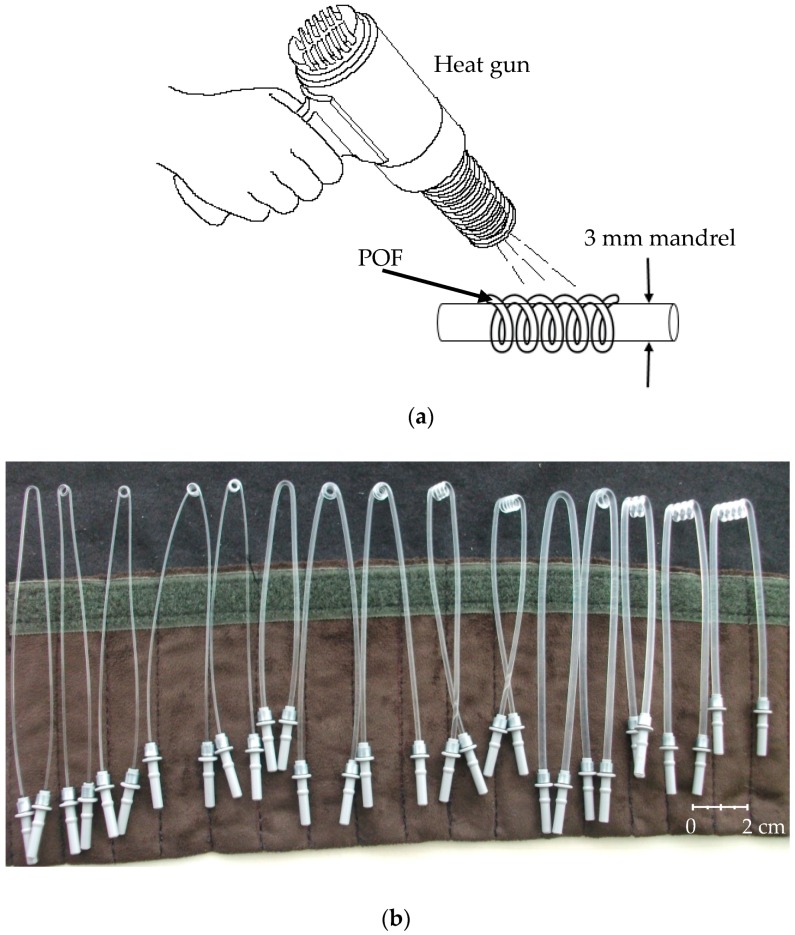
(**a**) Fabrication process of the probes; (**b**) Sensor probes.

Sensing probe is the most important part of the sensor system. We used three types of POFs having 1, 2, and 3 mm diameters to prepare the probes. In order to shape the sensing area, we heated the fibers up to the softening temperature and then we coiled them on a mandrel of 3 mm diameter. The fabrication process is shown in Figure 3a. Thus, we obtained different coils and hence different interaction lengths with the surrounding medium. The number of probes prepared in this manner is 15 and they are given in Figure 3b.

**Figure 4:**
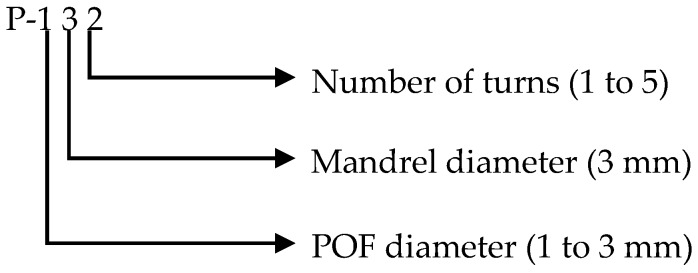
Explanation of the sensor probe coding used in this work.

Before the performance testing of the probes, we used a simple coding as given in Figure 4. For example, P-132 code represents a POF having 1 mm diameter, wound on a 3 mm mandrel by two turns, respectively.

### 2.3. Probe Selection

If we consider the designs from a simple point of view, the more interaction paths, the better sensitivity the sensor has. However, because of the escaping light, the increasing number of the turns limits the optical power reaching to the sensor output. On the other hand, another parameter known as normalized frequency of the fiber plays an important role on cladding power of the fiber [34]. The normalized frequency is given by [35]

(1) $$V=\frac{2\pi }{\lambda }a(\mathrm{NA})$$

where, λ is the vacuum wavelength of the optic source, *a* is the core radius, and NA is the numerical aperture of the fiber. Because of the fact that the wavelength and the NA are constants in our designs, the normalized frequency will vary with the core diameter. As a result, the cladding power fraction (η) determining the sensitivity of the sensor design will inversely depend on the normalized frequency and hence the core diameter in accordance with [35],

(2) $$\eta =\frac{P_{clad}}{P_{clad}+P_{core}}≅\frac{4}{3V}$$

In order to select the best candidates we performed some measurements by using the probes. During the measurements we recorded the readouts as I_0_ and I_1_ for which the probe is in air and in water, respectively. Since refractive index difference between the air (n_0_ = 1.00) and water (n_1_ = 1.33) gives sufficient outputs, we have simply used water. The results for determining the best candidates are given in Table 1. 

NOP means in Table 1 that the optical power at the input of the receiver is less than 0.135 mW because of the losses in the sensing region. So, the receiver does not produce electrical power. After the measurements, we can consider the probes coded by P-232, P-233, and P-331 as the best candidates. Also we can see from Table 1 how POF diameters can affect the sensor readouts. By keeping constant the bending radius and the number of turns, the readouts against the POF diameter can be summarized as given in Table 2. One can expect that waveguide modes are tightly confined as POF diameter increases in contrast to Equation (2). However, bending radius will decrease when POF diameter increases since the mandrel has a constant radius.

In accordance with Equation (1), the sensor readouts can be tuned by wavelength of the optical source and by refractive index differences between the POF cladding and the surrounding medium. Since the wavelength of the source and the refractive index of the cladding are constant in this work, we used other parameters for sensor tuning.

## 3. Results and Discussion

Some preservatives such as formaldehyde, hydrogen peroxide, and sodium carbonate, etc., are used to prevent acidity in milk [36]. In this work, we prepared different solutions by using commercial low-fat (0.1%) milk in which these preservatives were added in different concentrations. The refractive index of the milk increases up to 1.35093 with the preservatives added, while the initial value is 1.34550. It is well known that the refractive indices of the samples are highly dependent on the temperature. So we conducted the experiments between the temperatures of 24 and 26 °C in order to prevent index fluctuations in the samples. The measurements for the different concentrations of the preservatives are given in Table 3, Table 4, Table 5 and Table 6. The tables show the readouts of P-233 because it has better sensitivities. We did not use P-331 because it has a 3-mm-diameter POF which creates immeasurable losses due to over-bending. This situation points out, in our designs, that bent-dependent power losses will increase as the POF diameters get larger since the mandrel diameter is constant at 3 mm. A detailed discussion of the losses due to bending can be found in Reference [37]. Note that in all the sensor readouts in the tables, I_0_ and I_1_ are in arbitrary units; the readouts get higher when the refractive index of the surrounding medium increases. Also note that we used commercially available preservatives to adjust the refractive indices. For example, hydrogen peroxide-39 means that the commercial packaging contains 39 mL pure hydrogen peroxide and 61 mL distilled water. This simple explanation is valid for the other preservatives. 

As can be seen from the tables, the sensor probe P-233 can respond to very small changes in the concentration of as much as 1.24%, while the average is 1.43%. The sensitivity in terms of the refractive index difference has very interesting results. The maximum difference in the refractive index is 1.34612 − 1.34550 = 6.2 × 10^−4^ (in Table 6, rows 2 and 1) while the minimum value is 1.34686 − 1.34674 = 1.2 × 10^−4^ (in Table 3, rows 11 and 10). These numerical evaluations show that the sensor probe is able to detect 1.5% changes in concentrations or 5.0 × 10^−4^ changes in refractive indices, roughly. 

**Figure 5:**
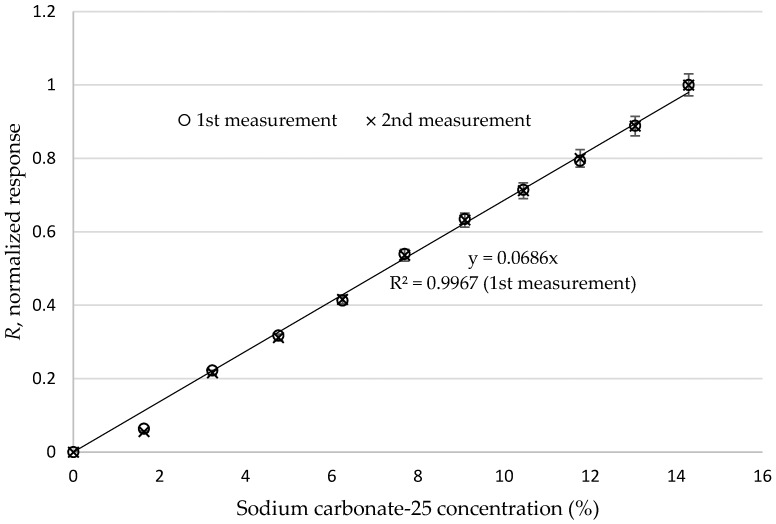
Normalized response of P-233 against percentage concentration (error bars represent less than 3% error for the measurements).

As another performance evaluation, we used a normalized response (*R*) as given below:

(3) $$R=\frac{x_{i}-x_{\min}}{x_{\max}-x_{\min}}$$

where *x_i_* refers to any of the I_0_−I_1_ values in Table 6 while *x_max_* and *x_min_* are the maximum and minimum values of the column, respectively. Then we depicted the response of P-233 for sodium carbonate-25 solutions in terms of *R* graphically in Figure 5. It can clearly be seen that there is an excellent linearity between the concentration and normalized response. In order to test the repeatability of the responses, we made an additional measurement. The errors for the measurements are under 3%. These results were shown with error bars in Figure 5.

**Figure 6:**
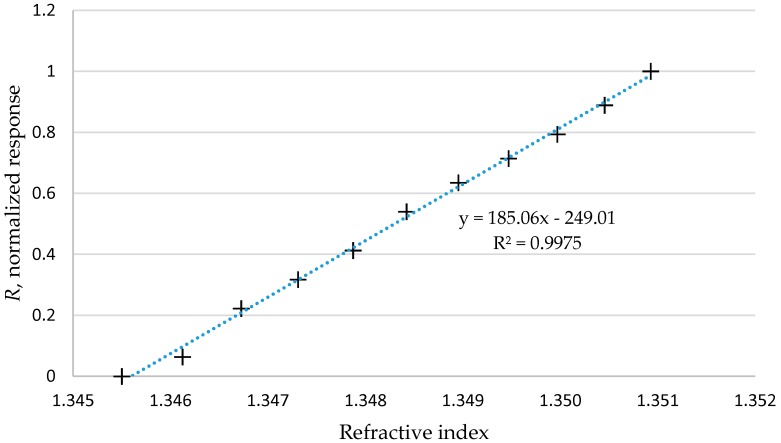
Normalized response of P-233 against refractive index change.

The sensor response of probe P-233 in terms of the refractive index change is shown in Figure 6. It can be clearly seen from the figure that there is an excellent linear relationship between the refractive indices and the sensor responses.

## 4. Conclusions

A fiber optic sensor made by bent plastic optical fibers has been presented. The sensor is sensitive to the refractive index changes determined by the concentrations of the medium surrounding the sensing area. The surrounding medium is low-fat milk, the refractive index of which is altered by some preservatives, i.e., formaldehyde, hydrogen peroxide, and sodium carbonate. By adding the preservatives, the resulting refractive indices are changed between 1.34550 and 1.35093 for the minimum (0%) and maximum (14.3%) concentrations of the sodium carbonate-25, respectively. The average change in the concentration is about 1.43%. As a result, we obtained excellent linearity between the concentration and normalized response of the sensor.

It is concluded that the sensor presented in this work can be adopted in liquid food processes to monitor for any target parameter subject to refractive index changes. 

## Figures and Tables

**Table 1:** Determining the best candidates for the sensor probes.

Probe	Readouts (in Arbitrary Units)		I_1_−I_0_
	I_0_ (in Air)	I_1_ (in Water)	
P-131	1000	1002	2
P-132	1000	1003	3
P-133	1000	1005	5
P-134	1000	1006	6
P-135	1003	1017	14
P-231	1002	1052	50
**P-232**	**1004**	**1113**	**109**
**P-233**	**1005**	**1258**	**253**
P-234	1022	NOP ^1^	-
P-235	1043	NOP	-
**P-331**	**1012**	**1303**	**291**
P-332	1048	NOP	-
P-333	1090	NOP	-
P-334	1298	NOP	-
P-335	1401	NOP	-

^1^ NOP: No optical power at the output.

**Table 2:** Effect of core diameters on sensitivity for the sensor probes.

Probe	Readouts (in Arbitrary Units)		I_1_−I_0_
	I_0_ (in Air)	I_1_ (in Water)	
P-131	1000	1002	2
P-231	1002	1052	50
P-331	1012	1303	291

**Table 3:** Sensor readouts with probe P-233 for hydrogen peroxide-39.

Hydrogen Peroxide-39 Concentration (%)	Refractive Index	I_0_ (in Air)	I_1_ (in Milk)	I_1_−I_0_
0.00000	1.34550	1008	1310	302
1.63934	1.34566	1008	1310	302
3.22581	1.34581	1008	1312	304
4.76190	1.34595	1008	1313	305
6.25000	1.34609	1008	1314	306
7.69231	1.34623	1008	1315	307
9.09091	1.34636	1008	1316	308
10.44776	1.34649	1008	1319	311
11.76471	1.34662	1008	1319	311
13.04348	1.34674	1008	1321	313
14.28571	1.34686	1008	1322	314

**Table 4:** Sensor readouts with probe P-233 for sodium carbonate-12.5.

Sodium Carbonate-12.5 Concentration (%)	Refractive Index	I_0_ (in Air)	I_1_ (in Milk)	I_1_−I_0_
0.00000	1.34550	1008	1310	302
1.63934	1.34571	1008	1314	306
3.22581	1.34591	1008	1318	310
4.76190	1.34611	1008	1322	314
6.25000	1.34630	1008	1324	316
7.69231	1.34648	1008	1326	318
9.09091	1.34666	1008	1328	320
10.44776	1.34684	1008	1330	322
11.76471	1.34701	1008	1330	322
13.04348	1.34717	1008	1331	323
14.28571	1.34733	1008	1332	324

**Table 5:** Sensor readouts with probe P-233 for formaldehyde-37.

Formaldehyde-37 Concentration (%)	Refractive Index	I_0_ (in Air)	I_1_ (in Milk)	I_1_−I_0_
0.00000	1.34550	1008	1310	302
1.63934	1.34598	1008	1312	304
3.22581	1.34644	1008	1318	310
4.76190	1.34689	1008	1320	312
6.25000	1.34732	1008	1323	315
7.69231	1.34774	1008	1329	321
9.09091	1.34815	1008	1334	326
10.44776	1.34854	1008	1336	328
11.76471	1.34892	1008	1339	331
13.04348	1.34930	1008	1343	335
14.28571	1.34966	1008	1346	338

**Table 6:** Sensor readouts with probe P-233 for sodium carbonate-25.

Sodium Carbonate-25 Concentration (%)	Refractive Index	I_0_ (in Air)	I_1_ (in Milk)	I_1_−I_0_
0.00000	1.34550	1008	1310	302
1.63934	1.34612	1008	1314	306
3.22581	1.34673	1008	1324	316
4.76190	1.34731	1008	1330	322
6.25000	1.34788	1008	1336	328
7.69231	1.34842	1008	1344	336
9.09091	1.34895	1008	1350	342
10.44776	1.34947	1008	1355	347
11.76471	1.34997	1008	1360	352
13.04348	1.35046	1008	1366	358
14.28571	1.35093	1008	1373	365
